# Surface Modification of Mesoporous Silica Nanoparticles for Application in Targeted Delivery Systems of Antitumour Drugs

**DOI:** 10.3390/polym16081105

**Published:** 2024-04-16

**Authors:** Svetlana Kovtareva, Lyazat Kusepova, Gaukhar Tazhkenova, Togzhan Mashan, Karlygash Bazarbaeva, Eldar Kopishev

**Affiliations:** Department of Chemistry, Faculty of Natural Sciences, L.N. Gumilyov Eurasian National University, Astana 010000, Kazakhstan; kovtareva_syu@enu.kz (S.K.); kussepova_la@enu.kz (L.K.); tazhkenova_gk@enu.kz (G.T.); mashan_tt@enu.kz (T.M.); bazarbayeva_kzh@enu.kz (K.B.)

**Keywords:** mesoporous silica, inorganic nanoparticles, drug delivery, anticancer drugs, surface modification, polymers

## Abstract

The problem of tumour therapy has attracted the attention of many researchers for many decades. One of the promising strategies for the development of new dosage forms to improve oncology treatment efficacy and minimise side effects is the development of nanoparticle-based targeted transport systems for anticancer drugs. Among inorganic nanoparticles, mesoporous silica deserves special attention due to its outstanding surface properties and drug-loading capability. This review analyses the various factors affecting the cytotoxicity, cellular uptake, and biocompatibility of mesoporous silica nanoparticles (MSNs), constituting a key aspect in the development of safe and effective drug delivery systems. Special attention is paid to technological approaches to chemically modifying MSNs to alter their surface properties. The stimuli that regulate drug release from nanoparticles are also discussed, contributing to the effective control of the delivery process in the body. The findings emphasise the importance of modifying MSNs with different surface functional groups, bio-recognisable molecules, and polymers for their potential use in anticancer drug delivery systems.

## 1. Introduction

There is a steady increase in the incidence of cancer worldwide. Despite significant progress in the fight against oncology, malignant neoplasms occupy the leading place among the causes of mortality. According to the World Health Organisation, about 10 million people worldwide died of cancer in 2020 [1]. Therefore, the development of more effective dosage forms remains an urgent task in the treatment of cancerous tumours.

The history of cancer treatment represents a long and evolving process in the development of medicine and science. In ancient times, cancer was often regarded as an incurable disease, and treatment was limited to symptomatic support and pain relief using herbs and plants. In the Middle Ages, with the development of surgery, surgical intervention began to be used to remove cancerous tumours, giving patients a chance of recovery. Later, the discovery of X-rays and the development of biopsies enabled more accurate cancer diagnosis and staging. In the mid-20th century, chemotherapy and radiation therapy were developed. These treatments became the standard in the fight against cancer, giving more patients a chance of recovery. But when targeting tumours, classical chemotherapy drugs also damage healthy cells, which causes undesirable side effects [2,3] and is one of the main causes of high mortality among cancer patients [4,5]. Radiotherapy, in turn, affects not only the tumour but also the surrounding healthy tissues, resulting in a variety of side effects [6,7]. In recent decades, research has led to the development of new treatments such as immunotherapy and molecularly targeted therapies. These methods make it possible to attack cancer cells more precisely and minimise side effects.

**Figure 1:**
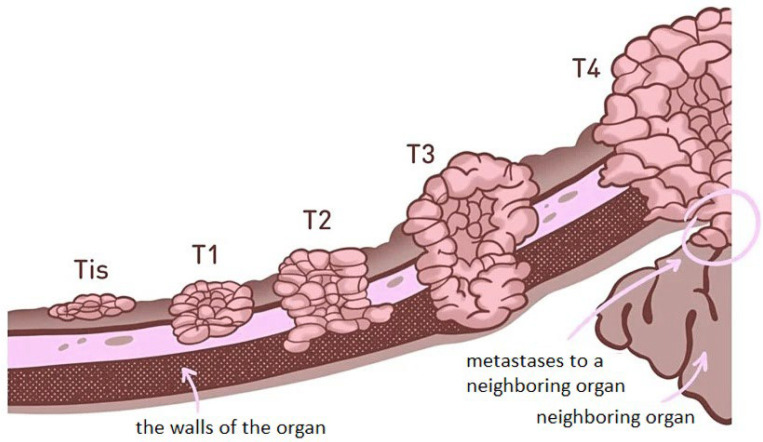
Stages of malignant tumour development. The positions Tis, T1, T2, T3, T4 shown in the figure correspond to the stages of tumor development stage 0, stage I, stage II, stage III, stage IV, respectively, which is also reflected in Table 1. (Reprinted with permission from the reference with changes [8]. Copyright © 1987, International Union Against Cancer Geneva.)

To date, a variety of methods for treating oncological diseases have been developed, and they are selected depending on the stage of the cancer process (Figure 1 and Table 1).

In the early stages, radical surgery is the mainstay of treatment [9,10]. Radiation [11,12] and chemotherapy [13,14] are used at different stages of cancer and can be combined with surgery. Immunotherapy is used in advanced stages of cancer [15,16]. Hormone therapy is used for hormone-sensitive cancers such as breast [17] or prostate cancer [18]. Targeted therapies, which act locally on specific molecules that are involved in the growth and development of cancer cells, have been approved for the treatment of many cancers [19]. For example, the drug ibrutinib was approved by the FDA (Food and Drug Administration) in 2013 for the treatment of mantle cell lymphoma (MCL) and chronic lymphocytic leukaemia (CLL) [20,21]. Ibrutinib targets the inhibition of Bruton’s tyrosine kinase, which is necessary for the normal functioning of B-lymphocytes. Thus, reducing the activity of this kinase with ibrutinib prevents the development of malignant B-cells such as MCL and CLL. This drug also disrupts the interaction of malignant cells with the surrounding microenvironment that ensures their viability. The choice of treatment method also depends on the type of cancer and the patient’s overall condition. Rarely, only one method is used; more often, complex therapy is performed.

Research for the fight against cancer is constantly ongoing. The development of directed transport systems for antitumour drugs is one of the most promising ways of creating new dosage forms, which can significantly improve drugs’ effectiveness and reduce their negative impact on the body. This approach is based on the fundamental differences between normal and cancer cells. Its aim is to improve the effectiveness of cancer treatment while reducing the occurrence of adverse reactions and side effects in patients. One key concept in the development of such systems is the delivery of drugs directly to the tumour, bypassing normal tissues. This approach increases the concentration of the drug in the tumour, contributing to the more effective destruction of cancer cells.

There are review articles on the potential of using mesoporous silica nanoparticles as delivery systems for various drugs [22,23,24]. Our review covers the analysis of studies conducted over the last 15 years and focuses on the surface modification of MSNs with polymers to improve their biocompatibility and cellular uptake, increase circulation time in the body, and prevent premature release, facilitating their application in the delivery of anticancer drugs directly to a tumour.

## 2. Application of Micro- and Nanoparticles in Drug Delivery Systems

Most of the research on the effectiveness of the targeted delivery of anticancer drugs focuses on the use of micro- and nanoparticles [25,26,27,28,29]. Nanoparticles have several unique features. Firstly, they have a developed specific surface area, which enables the high sorption capacity of nanoparticles. Secondly, they have physicochemical properties that allow them to penetrate cell membranes and cross the blood–brain barrier, constituting a difficult task for drug molecules [30].

The use of micro- and nanoparticles for the creation of new dosage forms allows solving such problems as those given below:

- Ensuring an optimal pharmacological effect [31,32];
- Allowing targeted transport and controlled release of the drug substance [32,33];
- Provoking minimal side effects [31,34];
- Ensuring convenience of administration [35].

In recent years, significant technological advances have been made in the field of cancer nanomedicine. Many developments are in the active stage of clinical trials, and some of them have already been applied in therapeutic practice [36,37]. Despite the potential efficacy of nanoscale drug carriers for use in cancer treatment, which has been demonstrated in studies at both preclinical and clinical stages, there are still a number of limitations that need to be addressed.

**Figure 2:**
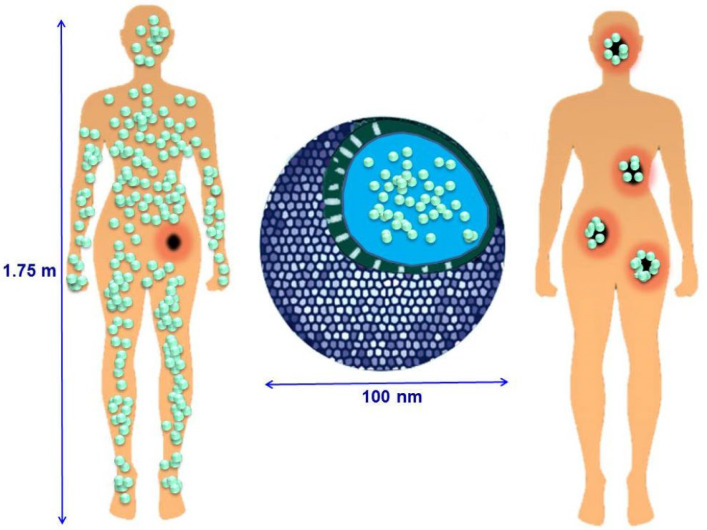
Schematic representation of drug applications for cancer treatment: conventional chemotherapy and nanomaterial-based targeting therapy. (Reprinted with permission from reference [38]. Copyright © 2015 Licensee MDPI, Basel, Switzerland.)

Targeted delivery involves the following mechanism: the carrier containing the drug enters the bloodstream, circulates through the body, and accumulates exclusively in the area of the lesion (Figure 2).

**Figure 3:**
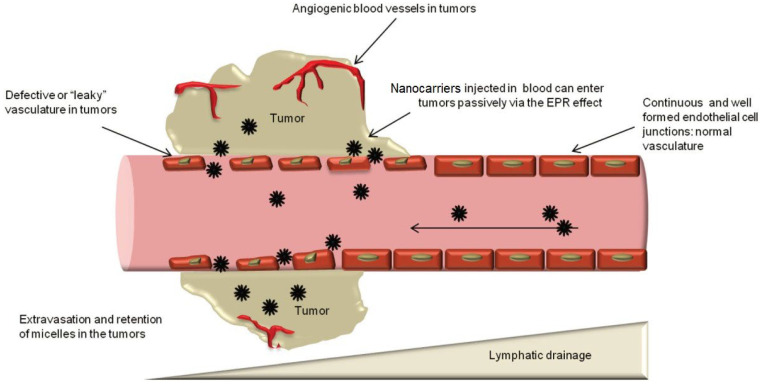
Enhanced permeability and retention (EPR) effect and passive targeting. (Reprinted with permission from reference [40] Copyright © 2014 Jhaveri and Torchilin).

The “enhanced permeability and retention” (EPR) effect, first described by I. Matsumura and H. Maeda over thirty-five years ago [39], can enhance drug accumulation at the tumour site (passive targeting). Their research demonstrated that the EPR effect is a result of excessive vascular overgrowth caused by a tumour’s need for oxygen and nutrition, leading to defects ranging up to 200 nm in diameter in the vessel walls. Nanoparticles can penetrate the tumour through these defects. Tumour growth causes compression of lymphatic vessels, preventing normal lymphatic outflow and promoting nanoparticle retention (Figure 3). Therefore, EPR-based drug delivery does not affect healthy tissues. However, the EPR effect is not specific to all cancers.

**Figure 4:**
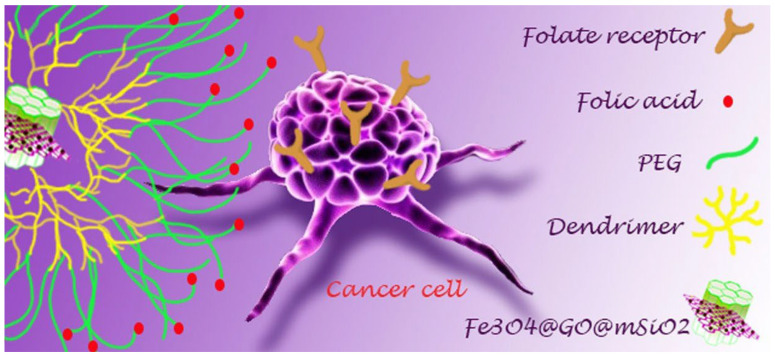
Schematic representation of active targeting of a drug nanocarrier and uptake by tumour cells. (Reprinted with permission from reference [41]).

Targeting a specific cell type is achievable via “molecular targeting” toward the surface (active targeting). For example, receptors for folic acid (FA) and transferrin (Tf) are present in significantly higher numbers on the surfaces of tumour cells compared to those on healthy cells. This fact allows the use of folic acid [41,42,43] or transferrin [44,45] as navigator molecules for the precise targeting of cancer cells (Figure 4).

**Figure 5:**
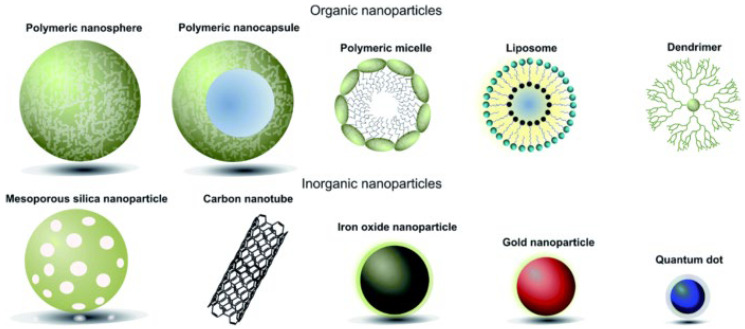
Graphical representation of different types of nanoparticles used in biomedical applications. (Reprinted with permission from reference [66]. Copyright © 2016, The Royal Society of Chemistry).

In addition to the ability to penetrate, specifically recognise, and bind to cancer cells, a drug nanocarrier needs to meet a number of requirements, including biocompatibility, a lack of toxicity, sufficient capacity and ease of drug loading, the ability to provide protection from the reticuloendothelial system (RES), and robustness of drug retention during delivery [46]. In micro- and nanoparticle-based delivery systems, drugs can be chemically bound to the transporter, dispersed as an emulsion in the transporter material, or encapsulated within it. Drug-released containers should not accumulate in the body. The route of entry into cancer cells is primarily determined by the material of the nanoparticle. Currently, the following nanoforms are used as nanoscale carriers for drugs (Figure 5):

- Biological and biogenic nanoparticles (enzymes, proteins, ribosomes, and viruses) [47,48,49];
- Polymer nanoparticles and nanostructures (polymer nanoparticles, polymer nanocapsules, polymer micelles, and dendrimers [50,51,52];
- Liposomes [53];
- Perfluorocarbon nanoparticles [54,55];
- Carbon nanoparticles (nanotubes, fullerenes, graphene, and nanodiamonds) [56,57,58];
- Inorganic nanoparticles (metals such as gold, silver, platinum, titanium, zinc, and iron; metal and nonmetal oxides; and magnetic nanoparticles) [59,60,61,62,63];
- Quantum dots and semiconductor nanocrystals [64,65].

**Figure 6:**
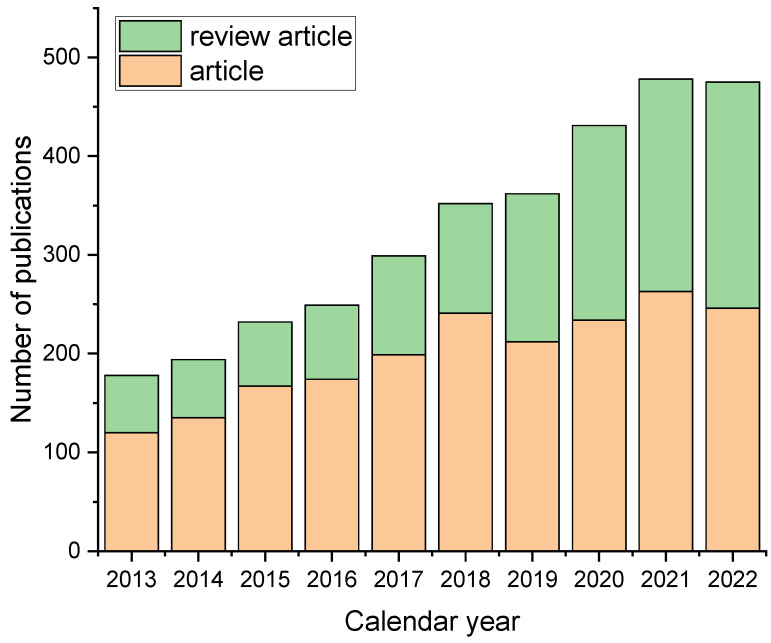
Analysis of the dynamics of publications on the use of inorganic nanoparticles for targeted drug delivery. Search was performed using the combination of the keywords “inorganic nanoparticles” and “drug delivery”.

Inorganic nanoparticles are some of the most widespread nanomaterials. Their amenability to use as drug carriers is due to their low toxicity, their ability to be excreted from the body, the fact that they do not accumulate in the liver, kidneys, spleen, and other organs. An analysis of the dynamics of publications, conducted on the search platform Web of Science from 2013 to 2022 using the combination of the keywords “inorganic nanoparticles” and “drug delivery,” showed an annual increase in the number of articles in this area (Figure 6). Scientists’ interest in using such nanostructures for targeted drug transport continues to grow as these structures offer unique opportunities for the more effective and safer treatment of many diseases. Among inorganic nanoparticles used for targeted drug delivery, magnetic nanoparticles, gold nanoparticles, silicon dioxide, calcium carbonate and phosphate, and titanium dioxide are the most in demand today.

## 3. Mesoporous Silicon Dioxide Is a Promising Nanocarrier for Drugs

**Figure 7:**
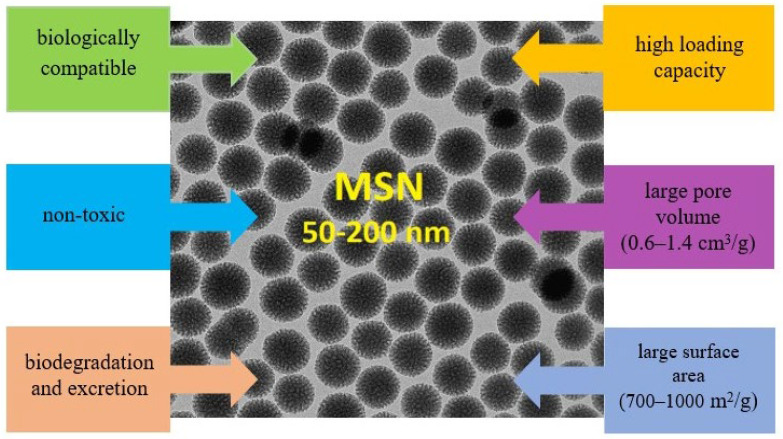
Main characteristics of mesoporous silica nanoparticles.

Mesoporous silica nanoparticles are promising nanoscale carriers due to the ease with which they can be synthesized and their homogeneous structure, tuneable size (50–200 nm) [67], and large pore volume (0.6–1.4 cm^3^/g) [68,69]. With a high specific surface area (700–1000 m^2^/g) [70,71], MSNs have high drug loading capacity. Nanostructured silica nanoparticles are non-toxic and biocompatible, capable of biodegradation in environments containing living organisms [72,73] (Figure 7).

**Figure 8:**
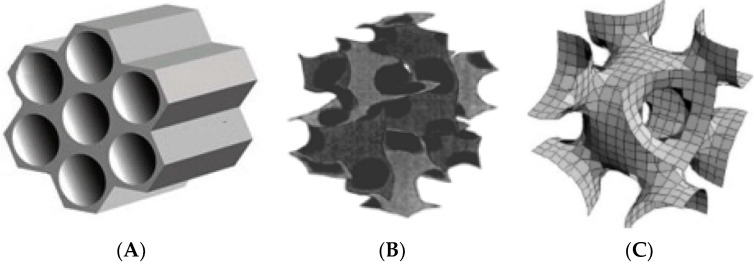
Schematic representation of the mesostructures of representative MSNs: (**A**) 2D hexagonal structures of MCM-41 and SBA-15 of the *p6mm* type. (Reprinted with permission from reference [74] Copyright © 2019 Licensee MDPI, Basel, Switzerland.) (**B**) Fragment of MCM-48 structure with *la3d* symmetry. (Reprinted with permission from reference [71].) (**C**) The 3D structure of the cubic-type *Im3m* SBA-16. (Reprinted with permission from references [78,80]. Copyright © 2016 International Journal of Pharmaceutical Investigation.)

Recently, great progress has been made in the synthesis and application of ordered MSNs with different structures. Most studies on the application of mesoporous silica nanoparticles for drug delivery mention MCM-41 (Mobil Composition of Matter, with a hexagonal two-dimensional *p6mm*-type pore structure with diameters ranging from 2 nm to 6.5 nm) [74,75], MCM-48 (possessing a three-dimensional cubic pore structure with *la3d* symmetry and an average diameter of about 2.7 nm) [71], SBA-15 (Santa Barbara Amorphous, with a hexagonally ordered array of cylindrical pores with an average diameter of 6 to 11 nm) [76,77], and SBA-16 (whose pore structure is a three-dimensional *Im3m* cubic cell with a diameter of 3 to 5 nm) [78,79] (Figure 8).

Mesoporous silica has been tested in many in vitro and in vivo studies and is considered safe by the FDA (Food and Drug Administration) [81]. However, several sources have reported that unmodified silica nanoparticles are toxic at high doses due to the interaction of surface silanols with cell membranes [82,83]. Additionally, unmodified MSNs exhibit poor ability for controlled drug release in response to internal or external stimuli such as pH, temperature, redox potential, a magnetic field, light, etc. Therefore, it is necessary to develop methods with which to modify the materials of MSNs to improve drug delivery to a specific pathological site, without drug leakage along the route, and allow rapid drug release at the site of action [84,85,86].

One of the most common methods of loading anticancer drugs into MSNs is the adsorption of the drug by mixing its solution with silica nanoparticles. The surfaces of unmodified MSNs are usually negatively charged due to the presence of a large number of hydroxyl groups and readily adsorb positively charged drugs. Functionalisation of the surface of MSNs can enhance electrostatic adsorption of drugs [87,88,89].

Currently, there are effective technological approaches to chemically modifying mesoporous silica nanoparticles to modify their surface properties [90,91,92]. MSNs can be modified with different surface functional groups [93,94,95] or coated with bio-recognisable molecules [96,97] and polymers [98,99] to further enhance biocompatibility and improve pharmacokinetics, biodistribution, and delivery of anticancer drugs to the tumour site.

## 4. Factors Affecting Cytotoxicity, Cellular Uptake, and Biocompatibility of Nanoparticles

**Figure 9:**
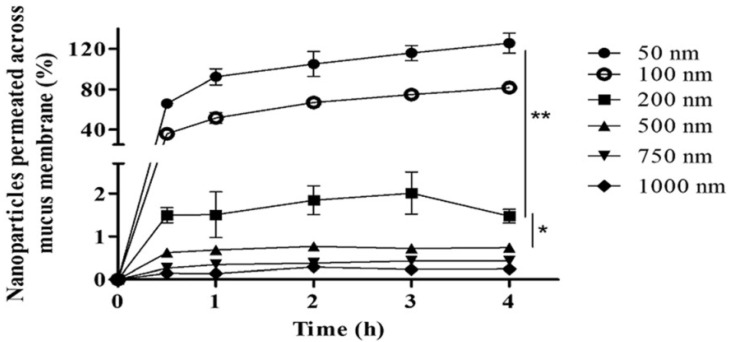
The effect of particle size on the percentage of nanoparticles penetrating the mucous membrane. * and ** mean that a value is significantly different at *p* < 0.05 and *p* < 0.01, respectively, compared to other sizes. (Reprinted with permission from reference [105].)

Determining the factors influencing the cytotoxicity, cellular uptake, and biocompatibility of nanoparticles is an urgent problem in the field of drug delivery. It is known that cytotoxicity and cellular uptake depend on the size of nanoparticles and the characteristics and functional properties of their surfaces. The surface properties and degree of aggregation of nanoparticles also determine their biocompatibility. Indeed, it has been reported that smaller particles induce significantly higher toxicity than larger ones in endothelial cells [100,101,102]. It has been shown in several studies that maximum cellular uptake is observed for 50 nm diameter particles [103,104,105] (Figure 9). The results obtained in [106] are consistent with the effective cellular uptake of 60 nm silica nanoparticles.

**Figure 10:**
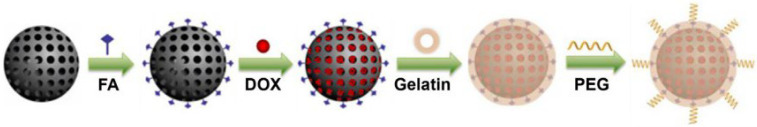
A scheme depicting the formation of a multifunctional doxorubicin delivery system based on MSNs that is functionalized with folic acid (FA) as a target ligand, coated with a layer of gelatin blocking DOX inside mesopores, and additionally decorated with polyethylene glycol (PEG) to increase circulation time in the body. (Reprinted with permission from reference [115] with changes.)

Size regulation can be useful for increasing the passive targeting of nanoparticles toward tumours, but their surface charges must also be taken into account [107]. Nanoparticles that have strong positive or negative charges are seen as an ‘alarm signal’ and are rapidly removed by the reticuloendothelial system, whereas particles with charges close to neutral have the ability to circulate and accumulate in a tumour for long periods of time. A proper polymer coating is required to ensure that there is a neutral charge on the surfaces of nanoparticles to increase their biocompatibility and circulation time in the body [81]. These affordances also solve problems such as preventing particle aggregation [108,109]. A commonly used strategy is the modification of the nanoparticle surface with polyethylene glycol (PEG) fragments (Figure 10). This process can be accomplished using two different techniques: covalent grafting or physical adsorption. PEG is a biocompatible polymer with very low immunogenicity and antigenicity and no toxicity. Polyethylene glycol forms a hydrophilic layer around particles with increased dispersibility, significantly increases the half-life of elimination from the bloodstream by delaying opsonisation, and improves the EPR effect [110,111]. By adding PEG of different molecular weights and concentrations, the thickness of the mesoporous shell can be adjusted [112]. However, pegylation has negative aspects, as it strongly inhibits cellular uptake and endosomal release, resulting in a significant loss of activity for the delivery system. Due to its high molecular weight at high doses, its accumulation in the liver is possible [113,114].

Despite significant advances in research, maintaining optimal therapeutic efficacy, i.e., preventing premature drug release into the bloodstream during rapid delivery to tumour tissue, remains a significant challenge for the development of targeted delivery systems. In the works of many authors, nanocontainers based on mesoporous silica with a “double coating” have been developed [43,116,117,118].

Meng H. et al. demonstrated that mesoporous silica nanoparticles loaded with doxorubicin (DOX), measuring 50 nm in size and coated with a copolymer of polyethyleneimine (PEI) and polyethylene glycol, can achieve a superior EPR effect compared to larger uncoated particles or 50 nm particles functionalized only with PEG [119]. Drug delivery efficiency was evaluated in vivo using a human xenograft tumour in nude mice after intravenous administration. Along with improved drug delivery, a significant reduction in side effects such as animal weight loss and decreased liver and kidney damage was observed. Hanafi-Bojd, M. et al. [120] and Pada, A.-K. et al. [109], in their studies, also showed that coating with PEG-PEI copolymer helps to prevent the aggregation of drug-loaded MSNs, reduce cytotoxicity, and enhance cellular uptake.

**Figure 11:**
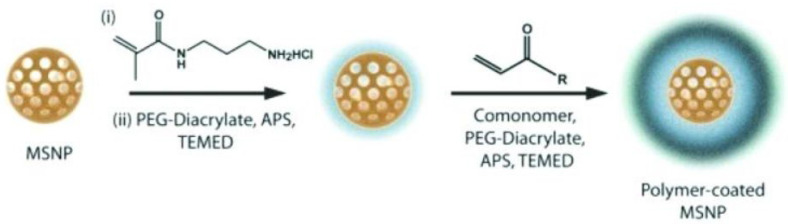
Scheme for preparation of covalently cross-linked polymer shell via radical polymerisation of monomers. (Reprinted with permission from reference [116]. Copyright © 2011 American Chemical Society).

A covalently cross-linked double-coated polymer shell was synthesised via the radical polymerisation of N-isopropylacrylamide (NIPAm) or polyethylene glycol diacrylate (PEGDA) monomers [116] (Figure 11). In the first step, the bifunctional molecule N-(3-aminopropyl) methacrylamide hydrochloride (APMA) was used to coat the anionic surface of MSNs. The amino group was electrostatically bound to the nanoparticle surface, while the acrylamide group was available for the radical polymerisation of monomers. Covalent crosslinking provides additional stability to the polymer shell. This coating deters the agglomeration of nanoparticles into larger aggregates (micrometre size) and provides longer circulation time and a high loading capacity of MSNs, the latter of which is an additional advantage of the proposed method.

A nanocarrier for gemcitabine delivery based on MCM-41, with a shell possessing a bilayer structure, was developed by Iranian scientists from the Sharif University of Technology [121]. The authors used a coating of a pH-sensitive polyacrylic acid-co-itaconic acid copolymer on the inside and human serum albumin (HSA) on the outside. Albumin was applied to the polymer layer through electrostatic interaction between the ammonium groups of the protein and the carboxylate ions of the copolymer shell. Albumin enhanced the biocompatibility and cellular uptake of the resulting carrier. A cytotoxicity assay of the drug-free nanocarrier using L929 mouse fibroblasts in vitro showed a level of cell viability >95%.

**Figure 12:**
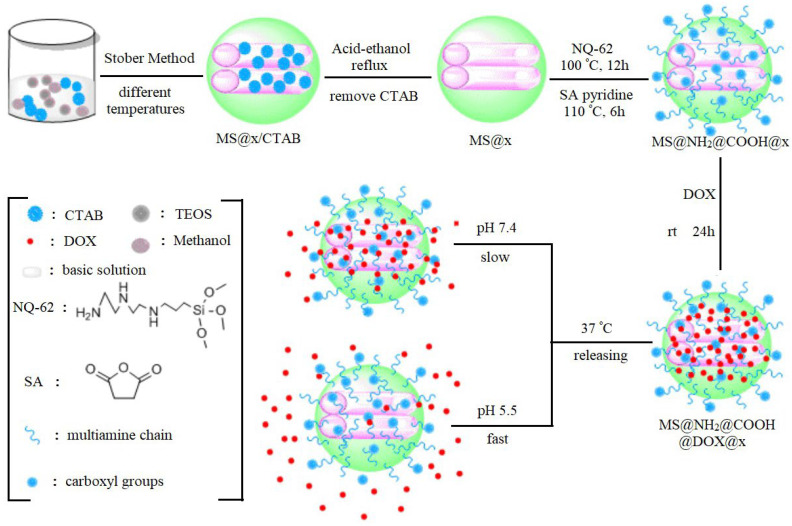
Schematic illustration of the synthesis strategy for organic functionalisation of MSNs. (Reprinted with permission from reference [122]. Copyright © 2019 World Scientific Publishing Company).

The right choice of functional groups and their concentrations can significantly influence the behaviour of nanoparticles in biological systems, determining their cytotoxicity and biocompatibility. The modification of MSNs with organic functional groups was demonstrated in a study by Chinese scientists [122] (Figure 12). Aminopropyl and carboxyl groups were grafted onto the surfaces of MSNs. The resulting MS@NH_2_@COOH materials exhibited minimal cytotoxicity toward A549 lung carcinoma cells. In contrast, the DOX-loaded nanomaterials (MS@NH_2_@COOH@DOX) exhibited a good killing effect against cancer cells, with a drug loading of 31.7%.

The cytotoxicity of cisplatin-loaded mesoporous silica nanoparticles functionalised with platinum (Pt) and carboxyl groups was investigated [123]. The nanocarriers were tested in vitro for the viability of three different cancer cell lines (A549, A2780, and MCF). The synthesised Pt/COOH-MSNs nanoparticles showed excellent antitumour efficacy, facilitating the cell death of 96.4% for A549, 84.2% for A2780, and 87.2% for MCF-7.

Active targeting significantly increases the efficiency of intracellular delivery, which allows the maintenance of high cytotoxicity towards a tumour but reduces cytotoxicity towards normal tissues. For example, a carrier made of mesoporous silica nanoparticles functionalized with folic acid (FA) was designed for the precise delivery of cisplatin to glioblastoma cancer cells [124]. Folic acid was used as a target molecule and was chemically attached to the surfaces of MSNs via carbodiimide reaction. Such carriers were highly biocompatible and enhanced the cytotoxic effect of the loaded cisplatin against LN 18 cells (human glioblastoma cells). The cellular uptake efficiency of folic-acid-coated mesoporous silica nanocomposites based on mesoporous silica for the targeted delivery of doxorubicin to cancer cells was also confirmed in Kumar, H. et al.’s work [125].

## 5. Stimuli That Control Drug Release

**Figure 13:**
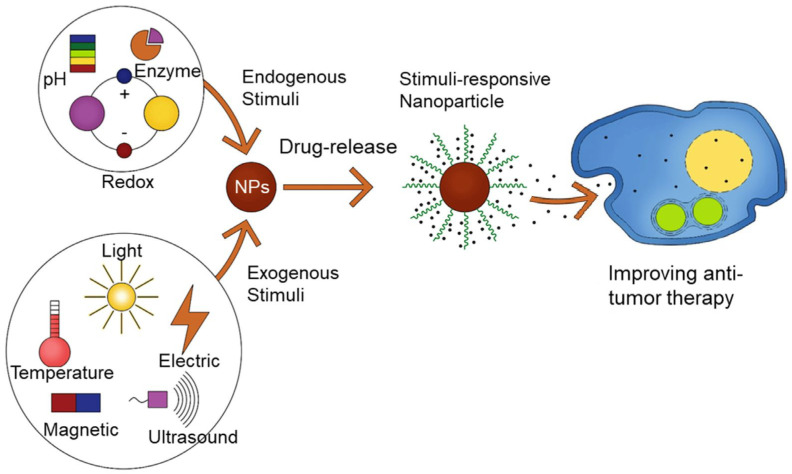
Stimuli controlling drug release from a nanocarrier. (Reprinted with permission from reference [126] with changes. Copyright © 2020 Licensee MDPI, Basel, Switzerland).

Delivery systems must control the release of a drug. The stimuli that regulate drug release can be diverse and include physical, chemical, biological, or combined factors. These include temperature, light, magnetic fields, pH, enzymes, redox potential, ultrasound, etc. (Figure 13).

### 5.1. Temperature- and pH-Sensitive Nanocarriers Based on Mesoporous Silica

**Figure 14:**
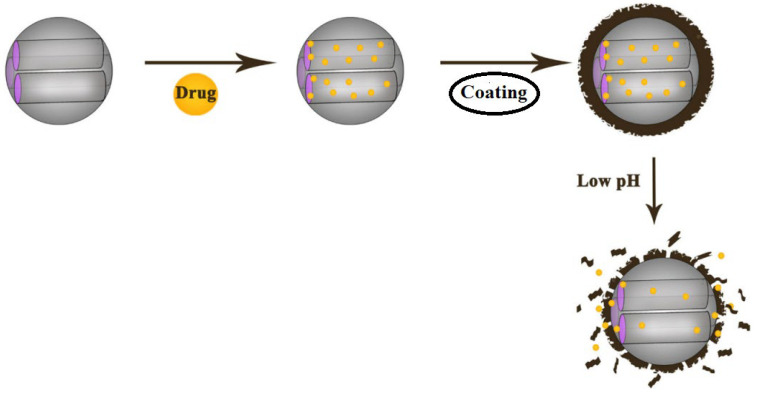
Preparation of pH-sensitive coated MSNs and drug release from nanocarriers. (Reprinted with permission from reference [133] with changes).

Typical internal stimuli used for the preferred release of drugs include pH and temperature. For example, most cancerous tissues have lower pH values (extracellular tumour pH ≈ 6.4–6.8, endosome pH ≈ 5.5, and lysosome pH ≈ 5.0) than healthy tissue and the bloodstream (pH ≈ 7.4) [127]. Applying a biocompatible polymer to the MSN surface to seal pores is a promising approach to creating desirable pH- and temperature-sensitive drug delivery systems [128,129,130,131,132]. The use of thermosensitive polymers allows for controlled drug release due to the fundamental differences in temperature between normal and cancerous tissue cells, allowing for tumour selectivity. Of particular interest are pH-sensitive polymer coatings on the surfaces of MSNs, which can more precisely control the rate, site of delivery, and release of active substances in target cells (Figure 14).

Thus, the authors of [127] coated magnetic mesoporous silica nanoparticles with PEGylated polyvinylpyridine (PEG-co-PVP). It was observed that the dissociation constant for polyvinylpyridine (pKa = 5.62) is in the range of the endosomal pH for cancer cells. Hence, this shell covers the surface of the nanoparticle and retains doxorubicin in the pores of mesoporous silica, but in an acidic medium at pH = 5.5, the electrostatic attraction is broken, leading to the collapse of the shell and the release of DOX from the nanopores.

In the work by Peng, H. et al. [134], a pH-sensitive MSN-PAA nanocarrier with a core of mesoporous silica nanoparticles and a shell of polyacrylic acid (PAA) was fabricated. The deposition of a pH-sensitive PAA polymer on MSNs was carried out via inoculum polymerisation. The drug salidroside was selected as a drug model. The in vitro results showed that PAA layers on the surfaces of MSNs can reversibly open and close at different pH values and thus regulate the uptake and release of salidroside from MSNs.

A number of studies have used a polymer shell that is sensitive to changes in the temperature and pH of an environment and obtained via the precipitation copolymerisation of N-isopropylacrylamide and methacrylic acid [135,136,137,138]. Mesoporous silica-based core–shell microspheres were designed to respond to a small temperature/pH difference between tumour tissue and healthy tissue under simulated physiological conditions. The elevated temperature and acidic pH, which are characteristic of the cancer cell microenvironment, lead to the shrinkage of the p(NIPAM-co-MAA) copolymer, resulting in the opening of pores, allowing controlled drug release. The results indicate that such DOX-loaded systems are efficiently taken up by cells under in vitro conditions and fully release the drug in an acidic intracellular environment. In an in vivo pharmacokinetics and biodistribution study, in tumour-bearing mice, the system DOX/MSN@NIPAM-co-MAA circulated in the bloodstream longer, with less accumulation in the heart and kidneys, compared to conventional MSN-DOX and had greater antitumour activity [136].

MSNs coatings consisting of pH-sensitive proteins can serve as a barrier regulating drug release from nanoparticles [139]. A drug delivery system based on sericin-coated MSNs for doxorubicin delivery (DOX@SMSNs) was developed. The sericin shell serves as a robust shield preventing the early release of encapsulated doxorubicin from MSN nanoparticles into the extracellular environment. The release of encapsulated doxorubicin is caused by the cleavage of sericin binding to the MSN surface in the acidic environment of lysosomes and simultaneously by lysosomal proteases that degrade the sericin shell.

In several works [74,140,141,142], the antitumour drug doxorubicin was encapsulated in the pores of mesoporous silica coated with gelatin (Gel) to investigate this drug’s pH-dependent controlled release behaviour and cytotoxicity. The results showed that this pH-sensitive MSN@Gel system was highly biocompatible and had remarkable drug loading behaviour. The gelatin coatings blocked the pore outlets of MSNs and retained encapsulated DOX under physiological conditions (pH 7.4). In contrast, under slightly acidic conditions at pH = 5.0–6.0, the system had an increased drug release rate. In vivo studies by Xu J.-H. et al. demonstrated that tumour growth in xenografted mice was significantly delayed without a noticeable loss of body weight, indicating a lower systemic toxicity of DOX/MSN@Gel compared to that of free DOX [74]. This suggests that MSN@Gel systems may be effective carriers of antitumour drug delivery systems.

Another pH-sensitive system was prepared based on polydopamine (PDA)-coated mesoporous silica nanoparticles via the oxidative self-polymerisation of dopamine in a neutral medium [133,143]. The PDA coating blocked the pores and retained doxorubicin inside the pores of MSNs under normal physiological conditions, which was useful for preventing premature release during circulation. In an acidic environment, the PDA coating was partially removed from the surfaces of MSNs, which was proved experimentally, so the DOX drug molecules could not be retained inside the pores of MSNs and released, which was useful for drug delivery in cancer treatment. These nanocarriers are characterised by their simple construction and easy synthesis. The authors of [144] also studied the in vitro behaviour of DOX@MSN-PDA and reported that DOX@MSN-PDA showed a slower release rate compared to DOX@MSN, probably due to the interfacial impermeable layer of PDA, which reduced the diffusion of DOX from MSN. In [145,146], polyethylene glycol was additionally grafted onto the PDA surface to enhance stability and biocompatibility under physiological conditions. The in vitro release profile of DOX/MSN@PDA-PEG demonstrated pH-dependent and gradual release of the drug. Studies of mesoporous silica nanoparticle systems with polydophamine-hyaluronic acid (PDA-HA) shells have been published on pH-sensitive release in tumours [147]. Hou, J. and colleagues [148] and Cheng, W. and co-authors [149] also evaluated the benefits of the MSN@PDA/DOX-PEG-FA system that is activated by folic acid and releases the drug at different pH values.

The technique often used by authors for applying polymer layers to the surface of a nanocarrier is the layer-by-layer (LBL) method. Thus, a strategy for the synthesis of functionalized nanocarriers based on MSNs with pH-dependent delivery characteristics and improved biosafety features has been proposed [150]. A multilayer polyelectrolyte coating of alginate and chitosan was prepared using the layer-by-layer (LBL) method. A study using HeLa cells revealed that the obtained nanocarriers had excellent biocompatibility and high cellular uptake efficiency and provided controlled drug release in acidic media. Xu X. and colleagues proposed the use of the layer-by-layer assembly technique (LBL) to close the pores of mesoporous silica with a biocompatible polyamidoamine dendrimer (PAMAM) and chondroitin sulfate (CS) [151]. Studies confirmed that the mesopores are effectively blocked at a neutral pH and open under acidic conditions. The coating gives the nanocarriers good dispersibility and blood compatibility. The developed nanocarriers are able to gradually release the active ingredient and thus reduce drug accumulation in major organs, potentially maximising the therapeutic effect while exerting minimal toxicity to healthy tissues.

### 5.2. Redox Drug Delivery

**Figure 15:**
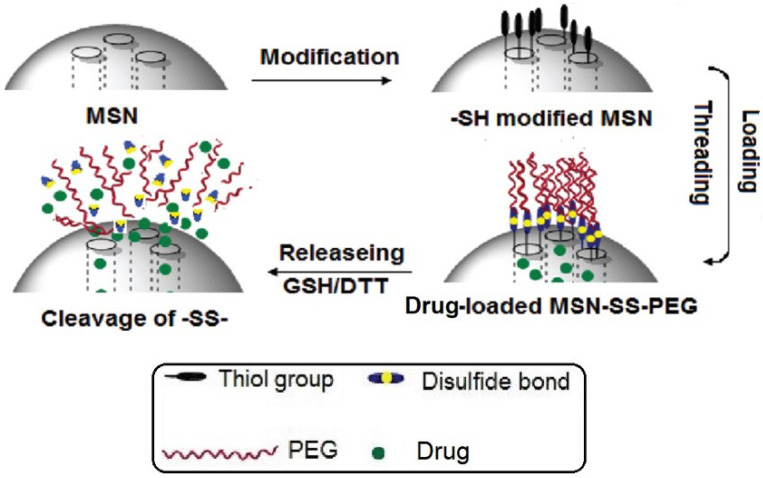
Illustration of the synthesis of redox-sensitive MSNs and drug release and disulfide cleavage. (Reprinted with permission from reference [156]. Copyright © 2012 American Chemical Society).

The shell of a nanosystem can break down or change its structure under the influence of oxidative and/or reductive processes, resulting in the controlled release of the drug substance at the right place and time. MSN-based systems capable of responding to glutathione (GSH) are common [152,153,154,155]. Glutathione is a tripeptide composed of the amino acids L-cysteine, L-glutamic acid, and glycine. It is ubiquitously present in the body and involved in important biological functions therein. Elevated levels of GSH in the tumour microenvironment cause it GSH repair any disulfide bond and convert to its oxidised form (GSSG). This property of GSH is utilised for the design of redox-sensitive drug carriers (Figure 15).

For example, the authors of [157] reported a nanocontainer for doxorubicin delivery in mesoporous MSNs, in which cytochrome (CytC) attached via a disulfide bond linker was used to germinate the pores. After entering the tumour, the disulfide bonds between MSNs and CytC are cleaved, thereby releasing the loaded drug. In a study by Yan, J. et al. [158], the anticancer drug paclitaxel (PTX) bound to mesoporous silica via a redox-sensitive disulfide bonding element that contributes to the loading efficiency, solubility, and stability of PTX. Drug release is controlled by redox reactions.

Researchers at Shanxi University, Taiyuan [159], have developed a nanocontainer for cancer spot chemotherapy based on mesoporous silica loaded with epirubicin and sequentially functionalized with bovine serum albumin (BSA) and folic acid (FA). Here, the BSA molecule serves as a redox-sensitive agent and prevents untimely drug leakage until the coating layer undergoes biological degradation in response to GSH exposure by breaking the disulfide bond in BSA. GSH-induced drug release can be controlled by adjusting the thickness of the polymer coating [160].

## 6. Antitumour Drugs in Mesoporous-Silica-Based Delivery Systems

The properties of mesoporous silica nanoparticles allow them to be used for the delivery of both water-soluble and -insoluble anticancer drugs.

A drug widely used in oncology for the treatment of various cancers is paclitaxel. The poor water solubility of paclitaxel makes its use difficult. Active research is underway regarding loading paclitaxel into the pores of MSNs, with the aim of optimising treatment, reducing side effects, and expanding its range of applications [161,162,163].

The standard drug of choice for pancreatic cancer monotherapy is gemcitabine (Gem). However, due to its poor pharmacokinetics, there is a need to develop new delivery systems for gemcitabine. In order to protect Gem from rapid metabolism in plasma, MSNs with grafted aminopropyl and carboxyethyl groups have been produced [164]. The encapsulation of gemcitabine in nanoparticles protects the molecule from degradation and premature elimination from the body. Saini, K., et al. [165] developed a carrier for gemcitabine based on mesoporous silica nanoparticles with diameters ranging from 42 to 64 nm to exploit the EPR effect. The particles with a pore diameter of 5.2 nm showed the best drug loading of 14.92% and the highest release of 58% at pH 5.5.

The most widely used drug for the treatment of a significant number of malignant neoplasms is the cytotoxic anthracycline antibiotic doxorubicin. One of the possible ways of reducing the toxicity of doxorubicin is the use of nanoscale transport systems for its transfer. Doxorubicin has intrinsic fluorescence in the red region of the spectrum (λ_exc_/λ_em_ 495/595 nm), so it is a convenient target for studying drug transport processes, including their intracellular penetration and release [166]. DOX is actively used as a model drug for evaluating drug loading and delivery using a variety of nanomaterials, including MSNs (Table 2).

The likelihood of killing cancer cells can be increased by using combination chemotherapy [181]. In this case, the nanocarrier contains synergistic pairs of chemotherapeutic drugs, allowing lower doses of each drug to be used, thereby reducing the toxicity and side effects of the treatment [182]. By selecting the optimal drug loading ratio, the best synergistic effect can be achieved. For example, in the aforementioned work [158], a group of scientists developed a carrier based on MSNs for the combined delivery of DOX and PTX with high selectivity between cancer cells and healthy breast cells. In this delivery system, PTX, using a disulfide-linked linker, was covalently attached to the surface of MSNs loaded with DOX. To control drug release under the acidic conditions of the tumour microenvironment, the obtained particles were electrostatically coated with polystyrene sulphonate. Thus, a dual pH- and redox-sensitive delivery system based on MSNs for the delivery of DOX and PTX was obtained. Another carrier providing a synergistic combination of Gem and PTX, based on lipid-coated mesoporous silica, was proposed by Meng, H. et al. [183]. Combined delivery, using MSNs, showed significantly higher efficacy in suppressing pancreatic cancer than drug mixtures or monotherapy.

There are studies on loading chemotherapeutic drugs such as cisplatin [184,185], sorafenib [186,187], temozolomide [188,189], 5-fluorouracil [190,191,192], irinotecan [193,194], and epirubicin [120,159]. This is just a small list of examples of anticancer drugs that can be encapsulated in nanoparticle MSNs for cancer treatment. This approach continues to be developed and investigated to improve the efficacy and safety of chemotherapy and other cancer treatments.

## 7. Conclusions

Mesoporous silica nanostructures are promising drug carriers. They possess many desirable properties, such as a large surface area, tuneable particle size and morphology, and easy surface functionalisation. However, despite the significant potential of MSNs in the treatment of tumours and other diseases, the understanding of their behaviour in the human body remains limited. The lack of data on the long-term effects of MSNs on the body is a significant barrier to moving this technology to a broader level of clinical application. It is necessary to establish a better understanding of the mechanism of mesoporous silica degradation in vivo and investigate the consequences of long-term use of MSNs as drug carriers.

Another important issue directly affecting MSNs’ efficacy and safety for patients is the increased drug load in nanoparticles. The higher the drug content in the nanocarrier, the lower the accumulation of silica in body tissues. There is a lack of information on the physicochemical patterns of interaction of MSNs with drugs. A deeper study of their interaction mechanism will make it possible to control the drug loading level and drug release, which is very important for the development and optimisation of drug delivery strategies based on MSNs.

Unlike other nanocarriers, the fabrication of MSNs is a simple and cost-effective process. Importantly, the functionalisation of the nanoparticle surface is of key importance in the context of developing effective delivery systems for anticancer agents. Various surface modification options allow the design of MSNs with a controlled mechanism of drug release under the influence of various stimuli such as changes in pH, temperature, or the presence of certain molecules. By modifying the surfaces of mesoporous silica nanoparticles, several important goals can be achieved:

- Improving biocompatibility;
- Increasing the ability of nanoparticles to retain drugs;
- Increasing the specificity of delivery;
- Allowing controlled drug release.

This research holds great potential for the development of intelligent drug delivery systems that can respond to specific conditions in the body and provide optimal treatment efficacy with minimal side effects.

In addition, various ligands can be attached to the surfaces of MSNs, allowing them to be used for disease detection and diagnosis. This is particularly important for the early detection of cancer. MSNs offer a wide range of promising affordances in the field of theranostics, which involves the integration of diagnostic and therapeutic capabilities in a single system. This facilitates a more effective and personalised approach to patient care.

Overall, understanding all the above aspects will help developers and researchers to better utilise the potential of MSNs as drug carriers and overcome the current limitations regarding their clinical use.

Undoubtedly, an ideal nanotransporter for the delivery of pharmacological drugs should not only provide effective functional characteristics but also have high manufacturability in the production process. Only when these requirements are met will there be real prospects for the successful commercialisation of this product and its introduction into clinical practice.

## Figures and Tables

**Table 1:** Classification by stage of tumour development.

Stage of Development	Stage 0 (Tis)	Stage I (T1)	Stage II (T2)	Stage III (T3)	Stage IV (T4)
Localisation	Cancer is in place (in situ), does not manifest itself in any way, does not form vessels for its supply	Tumour measures up to 2 cm, localised, not extending outside the wall	2 to 5 cm tumour, extends beyond the wall, lymphatic involvement	Tumour measures more than 5 cm, is growing into surrounding tissues, multiple lymph node involvement	Tumour of any size, locally spreading, sprouting into surrounding tissues and organs
Surgical option	not recommended	operable	operable	mission-capable	inoperable
Metastasis	none	none	none	none	present
Forecast	favourable	favourable	treatable	treatable	incurable

**Table 2:** Types of delivery systems for the anticancer drug doxorubicin based on mesoporous silica.

Surface Modification	Method of Coating/Functionalisation	Study Model/Release	In Vitro/In Vivo Evaluation	Ref.
Polyethyleneimine and polyethylene glycol copolymer (co-PEI-PEG)	Graft copolymerisation	Human carcinoma xenograft in nude mice after intravenous injection/-	-/Reduced particle opsonisation, improved doxorubicin delivery to tumour xenograft site, reduced side effects	[119]
Poly-N-isopropylacrylamide—polyethylene glycol diacrylate (pNIPAm-co-PEGDA)	Surface radical polymerisation	Mouse models of subcutaneous human sarcoma xenograft/Release triggered by endogenous protease	-/Offers colloidal stability, temperature sensitivity, prolonged circulation in the blood, high loading capacity, and customisable release	[116]
Poly-N-isopropylacrylamide—methacrylic acid p(NIPAm-co-MAA)	Precipitation copolymerisation method	Model buffer solutions; mice carrying murine sarcoma cell line S-180/Sensitive to pH change	Great antitumour activity. DOX release at pH = 5.0 85.2% ± 4.8 for 48 h and 12.9% ± 2.2 at pH = 7.4/Significantly increased the duration of drug circulation and decreased DOX accumulation in the heart	[136]
Poly-N-isopropylacrylamide—methacrylic acid p(NIPAm-co-MAA)	Precipitation copolymerisation method	Phosphate buffer (PBS) with different pH values (7.4 and 5.0) and human cells (HeLa)/Sensitive to temperature and pH changes	At pH 7.4, 9–12% of DOX was released in 8 h at 37 °C or 50 °C. At pH 5.0, the release was 19% at 37 °C and 44% at 50 °C for 8 h. HeLa cells exhibited low cytotoxicity and efficient cellular uptake of MMSN@P(NIPAM-co-MAA) nanoparticles when incubated for 4 h and 48 h/-	[137]
Sericin (Ser)	Covalent envelopment (cross-linking with glutaric aldehyde)	Phosphate buffer (PBS) with different pH values, human cells (HeLa (liver cancer), HepG2 (hepatocytic carcinoma) and MCF-7 (breast cancer), female BALB/c mice /Sensitive to pH change	The loading efficiency of DOX was 29.1%. DOX release rates: 16.4% and 24.1% at pH 7.4, and 6.5, respectively, for 72 h. Under acidic conditions (pH 5.0), 53.9% was released within 72 h/No significant cardiac damage or degeneration were observed in mice treated with DOX@SMSNs	[139]
Gelatin (Gel)	Formation of the coating layer through adsorption, followed by crosslinking with glutaric aldehyde	Hep-G2 cells, model buffer solutions, xenografted mice/Sensitive to pH change	Good biocompatibility and efficient intracellular drug release. Release rates: approximately 18%, 44%, 54% and 83% of the drug within 440 min at pH 6.0, 5.0, 4.0 and 2.0 respectively/Tumour growth in mice was significantly inhibited without marked reduction in body weight	[74,140]
Polidophamine (PDA)	Oxidative self-polymerisation	Phosphate and acetate buffer solutions for simulating normal physiological conditions and intracellular conditions of cancer cells/Sensitive to pH changes	Under normal physiological conditions (pH 7.4), no detectable release of DOX was observed. In acidic solutions (pH 5.0, 4.0, 3.0), the release rate increased as the acidity increased. At pH 4.4, 60% of DOX was released within 72 h/-	[133,167]
Pegelated polydophamine modified with folic acid	Absorption, self-polymerisation	PBS buffer, 4T1 cells (breast cancer cells), eight-week-old female BALB/c mice/Sensitive to pH change	DOX loading efficiency is up to (35.43 ± 0.59%). DOX release: 80% release at pH 5.0 vs. 20% release at pH 7.4/Effectively accumulates in 4T1 tumour and demonstrates superior tumour inhibition effect	[148,149]
Two bilayers alginate/chitosan	Layer-by-layer assembly method (LbL)	Model buffer solutions (acetate, phosphate) and HeLa cells/Sensitive to pH change	Throughout the time period, approximately 10.7%, 48.6%, and 60.1% of DOX was released at pH 6.8, 5.2, and 4.0, respectively. The nanocarriers exhibited sustained intracellular DOX release and prolonged retention of DOX in the nucleus/-	[150]
Polyamidoamine (PAMAM) dendrimers and chondroitin sulphate (CS)	Layer-by-layer assembly method (LbL)	Phosphate-buffered saline (PBS)/Sensitive to pH change	Slow and sustained release of DOX and CUR at neutral pH, much faster in an acidic environment (pH = 3), 35% DOX and 17% CUR released within 28 h/-	[151]
Chitosan-polymethacrylic acid (CS-PMAA)	In situ polymerisation	HeLa cells/Sensitive to pH change	The release rate when the pH was lowered to 5.5 reached 70 wt.% after 24 h, which is almost four times higher than at pH 7.4/-	[168,169]
Poly N-vinylcaprolactam-methacrylic acid p(VCL- co -MAA)	Precipitation copolymerisation method	Model buffer solutions/Sensitive to temperature and pH changes	DOX release: 5.4% of drug in buffer with pH 7.4 within 24 h, at pH 6.5 and pH 5.0, 34.1% and 64.2%, respectively/-	[170]
Oligo ethylene glycol acrylate—N,N′ -cystamine bismethacrylamide poly(OEGA-co-CBMA)	Grafting of crosslinked copolymer	Phosphate buffered saline PBS + dithiothreitol DTT/By reduction-oxidation reactions	About 32% of the loaded DOX was released in pure PBS after 25 h. In PBS DTT solution (20 × 10^−3^ M), 85% of the drug was released after 10 h/-	[171]
Polyglycerol methacrylate (PGOHMA) and cucurbituryl (CB)	Self-assembly technology layer—by —layer (LbL)	Model buffer solutions, BALB/c nude mouse models/Sensitive to pH change	DOX molecules are tightly held inside the nanopores at pH = 7.4. When the pH is lowered to 5, the interaction between CB and PGOHMA layers weakens, and DOX is released/Showed high inhibition of tumour growth by 63% on day 28	[172]
Copolymer of dimethylamino-ethyl acrylate and polyethylene glycol methacrylate Poly(DMAEA-co-PEGMA)	Use of a combined “RAFT” polymerisation and “Graft From” strategy	Hela cell xenografts in nude mice, Model buffer solutions/Sensitive to pH change	Rapid drug release when soaked in acidic solution (pH 5.5) Soaking in acidic solution (pH 5.5)/Significantly increased EPR effect and tumour growth inhibition rate of 68.7%	[173]
Diblock copolymers of polyethylene oxide and L-lactide (Poly(EO-co-LLA))	Block copolymer grafting	Model buffer solutions/Sensitive to temperature and pH changes	Release at 45 °C is easier than at 25 °C. Reaching the maximum release (92.7%) at pH 4 takes only 24 h. When the pH value is increased to 7.4., the release decreases to 38.0%/-	[174]
Poly N-succinimidylacrylate (PSA)	Grafting with an acetal linker	Model buffer solutions (acetate, phosphate), HepG2 cell line/Sensitive to pH change	The cumulative amount of DOX released is up to 37.9% at pH 6.5, 78.4% at pH 5.0, and 93.5% at pH 2.0. Capable of targeting accumulation in cancer cells and effectively destroying cancer cells/-	[128]
Polyethylene glycol-co-polyvinylpyridine (PEG-co-PVP)	Through electrostatic interaction with grafted carboxylate groups	Model buffer solutions (acetate, phosphate)/Sensitive to pH change	At neutral pH (pH = 7.4), about 34% of DOX is released within 72 h. A change in pH = 5.5 results in increased release (about 75% within 72 h)/-	[175]
Boltorn H40 polyester, amine-functionalised polyethylene glycol (PEG-H40)	Grafting of H40 and modification with tertiary amine and PEG via chemical bond formation	Model buffer solutions, MCF-7 cells, male Balb/c mice /Sensitive to pH change	Payload efficiency: 36.5%; capture efficiency: 57.4%. DOX release at pH 7.4 and pH 6.8 was only 9.7% and 20%, respectively, after 48 h, and increased to 49% at pH 5.5/Very low cytotoxicity in mouse kidneys and liver and excellent biocompatibility with blood	[176]
Polyacrylamide (PAA)	Graft polymerisation method	Aqueous solution of nanoparticles containing doxorubicin/Sensitive to temperature change	Released 11.5 ± 2.4% in aqueous solution at 37 °C after 30 min and 67.6 ± 2.5% at 60 °C/-	[177]
Poly (2-diethylamino- ethyl methacrylate) (PDEAEMA)	Surface-initiated radical polymerisation with atom transfer (ATRP)	Model buffer solutions (phosphate, acetate), HeLa cells/sensitive to pH change, recovery, and light	At pH 7.4, less than 12% of DOX is released within 22 h, and at pH 5.0, about 64% of DOX is released. After pH 5.0 treatment with dithiothreitol (DTT), the most complete release of DOX occurs, enhanced by UV irradiation/-	[178]
Polyaspartic acid (PAsA)	Conjugation on the surface of MSNs via amide bonding	PBS with different pH values, HepG2 cells/sensitive to pH changes	DOX@P-MSNs were efficiently internalised by HepG2 cells, with 73% inhibition of cell growth and only 30% and 33% inhibition of cell growth with free DOX and DOX@MSNs. 10% of DOX was released at pH 7.4 and almost 56% at pH 4.5/-	[179]
Folic acid (FA)	Conjugation on the surface of MSNs via amide bonding	Model buffer solutions, ZR-75-1 and T47-D cell lines/sensitive to pH change	The loading efficiency of DOX is about 68%. At pH 7.4 < 20% and at pH 5.5 about 45% of DOX is released within 24 h. Empty MSN-FA shows no cytotoxicity, DOX@MSN-FA is significantly more effective in inducing cell death than DOX solution at different concentrations/-	[180]

## Data Availability

The original contributions presented in the study are included in the article. Further inquiries can be directed to the corresponding authors.
